# Comparative Analysis of Roller Milling Strategies on Wheat Flour Physicochemical Properties and Their Implications for Microwave Freeze-Dried Instant Noodles

**DOI:** 10.3390/foods14162885

**Published:** 2025-08-20

**Authors:** Junliang Chen, Peijie Zhang, Linlin Li, Tongxiang Yang, Weiwei Cao, Wenchao Liu, Xu Duan, Guangyue Ren

**Affiliations:** 1 College of Food and Bioengineering, Henan University of Science and Technology, Luoyang 471000, China; chenjl2020@haust.edu.cn (J.C.); 13849433924@163.com (P.Z.); txyamy@163.com (T.Y.); caoweiwei@haust.edu.cn (W.C.); wen_chaoliu@163.com (W.L.); duanxu_dx@163.com (X.D.); 2 Henan Province Engineering Research Center of Agricultural Products Processing Equipment, Luoyang 471000, China; 3 Henan Province Engineering Technology Research Center of Agricultural Product Drying Equipment, Luoyang 471000, China

**Keywords:** roll gap, break milling, sifting, particle size, in vitro digestibility

## Abstract

The milling process is a critical technological step that regulates wheat flour characteristics and ultimately determines end-product quality. This study systematically evaluated the effects of three key milling parameter adjustments in a laboratory-scale roller mill—double sifting (2S), double break milling (2BM), and increased roll gap (IRG)—on the physicochemical properties of wheat flour and the quality of microwave freeze-dried non-fried instant noodles. The results demonstrated that milling processes significantly influenced the particle size and composition of flour. The 2BM-IRG process increased the volume mean diameter of flour to 86.38 μm, while significantly improving flour extraction rate (69.80%), protein content (10.98%), and ash content (0.54%). In contrast, the 2S process significantly reduced the volume mean diameter (65.27 μm). These changes in flour properties directly affected noodle quality—noodles made from 2BM-IRG flour exhibited the highest rehydration ratio but also the greatest cooking loss, along with the lowest expected glycaemic index (eGI); noodles produced from 2S flour showed the highest hardness, while the 2BM process endowed noodles with superior elasticity. A correlation analysis revealed that the digestibility characteristics of noodles (eGI) were predominantly and significantly influenced by flour protein and ash content (*p* < 0.01), while also being significantly affected by particle size (*p* < 0.05). The study confirmed distinct quality trade-offs between different milling strategies. Therefore, by optimizing combinations of break milling and sifting processes, it is possible to develop specialized flour tailored for specific quality requirements.

## 1. Introduction

Globally, instant noodle consumption reached 123.1 billion servings in 2024, with China and Hong Kong ranking first with 43.8 billion servings (35.6% share). Key Southeast Asian markets included Indonesia (14.68 billion), India (8.32 billion), and Vietnam (8.14 billion), while the U.S. consumed 5.15 billion servings in North America [1]. As a ready-to-eat food, instant noodles continue to be favored by consumers around the world for their distinctive flavors and convenience. However, with the growing awareness of healthy eating, consumers are increasingly concerned about the health implications of traditional fried instant noodles. These concerns primarily stem from two health risks. Firstly, the frying process results in an excessively high fat content; secondly, high-temperature processing may generate harmful substances [2]. To address these issues, non-fried instant noodles (NFINs) have emerged as an improved alternative, utilizing low-temperature processing technology to reduce fat content while better preserving the nutritional value of ingredients [3]. However, it should be noted that current mainstream NFINs still use refined wheat flour as a raw material, resulting in a lack of dietary fiber and a high glycemic index, which fails to align with modern health-conscious dietary preferences.

To address these nutritional limitations, researchers have explored two primary approaches—incorporating alternative grains or bran additives, and optimizing native wheat flour properties through milling process modifications. By incorporating bran additives (e.g., wheat bran [4] or barley bran [5]) or alternative grain ingredients (e.g., quinoa flour [6] or barley flour [6]) as supplementary components into refined wheat flour (RWF), the noodle nutrition can be enhanced through increased dietary fiber and bioactive compounds. However, such formulations often require additional processing aids or compromised dough functionality. In contrast, strategically adjusting milling parameters (e.g., tempering treatment and sifting duration) can directly regulate flour composition and characteristics, achieving a synergistic improvement in nutrition and processing performance from the raw material source. In previous studies relating to milling technologies, Pallavi et al. [7] compared the differences in vitamin D3 fortification effects of wheat flour produced by hammer milling versus roller milling using different grinding processes. Tian et al. [8] evaluated the impacts of impact milling and friction milling on wheat flour properties. However, these studies primarily focused on the influence of equipment type. There is a lack of research regarding the preparation of wheat flours with varying processing degrees through regulating the key milling process parameters—including sifting times, bran milling times, and roller spacing—and particularly concerning their effects on the quality characteristics of NFINs.

Traditional NFINs still exhibit notable drawbacks—hot-air-dried products suffer from structural collapse and poor rehydration properties, while freeze-drying is energy-intensive and yields products with a loose texture [9]. Microwave freeze-drying (MFD) technology has demonstrated significant potential in food processing due to its distinctive advantages in high dehydration efficiency, excellent porous structure preservation, and superior nutrient retention. It has been widely applied in the drying of fruits [10], vegetables [11], edible fungi [12], and starch-based foods [13], yielding high-quality dehydrated products with freeze-drying-like characteristics. Although previous studies have investigated the effects of MFD process parameters on the quality of NFINs [14], research on the synergistic effects between wheat flour processing degree and MFD technology remains notably insufficient.

This study aims to evaluate the effects of wheat flours with different processing degrees by modulating key milling parameters (including sifting times, break milling times, and roll gaps) on the quality of microwave freeze-dried NFINs. The research focuses on elucidating the influence of compositional changes induced by processing on critical product quality characteristics (including color, texture, rehydration properties, water state and distribution, microstructure, sensory attributes, and in vitro digestibility), aiming to provide both a theoretical foundation and technical guidance for developing high-quality non-refined flour-based foods.

## 2. Materials and Methods

### 2.1. Materials

The wheat (variety: Hard Red Winter), originating from Nanyang City, Henan Province, China, was provided by Dengzhou Direct Reserve Depot Co., Ltd. of China Grain Reserves Corporation. The moisture content of the wheat was determined to be 0.10 ± 0.01 g/g (dry basis) according to AACC Method 44-15.02 [15].

### 2.2. Sample Preparation

Prior to milling, wheat kernels underwent cleaning, washing, drying, and conditioning treatments. First, impurities and moldy grains were manually removed from the wheat. The wheat was then rinsed three times with distilled water, before being drained. The cleaned wheat was dried in a 50 °C oven until the moisture content reached 12–14% (turned every 15 min). Finally, conditioning (tempering) was performed according to the NY/T 1094.1-2006 standard [16]. The required amount of water to achieve a target moisture content of 16% was calculated and was added by spraying. The wheat was tempered in a sealed container at 20–25 °C and 60–70% relative humidity for 24 h, with turning every 8 h, until the endosperm showed no hard core and the moisture content reached 16 ± 0.5%.

The experimental milling system (PLM-T model, Pekon Science & Technology Co., Ltd., Hangzhou, China) primarily consisted of two subsystems—a break system and a reduction system. Both systems employed a “milling–sifting” cyclic process. The break system was equipped with three pairs of corrugated rolls arranged in a dull-to-dull (D-D) disposition, operating at a roll speed ratio of 2.5:1. The dynamically adjustable roll gaps were precisely controlled by standard feeler gauges, implemented in two stages—0.5 mm for initial coarse crushing, followed by 0.05 mm for fine stripping. The reduction system utilized three pairs of sandblasted smooth rolls with a speed ratio of 1.25:1, featuring roll gaps of 0.05 mm and 0.03 mm for endosperm refinement.

**Figure 1:**
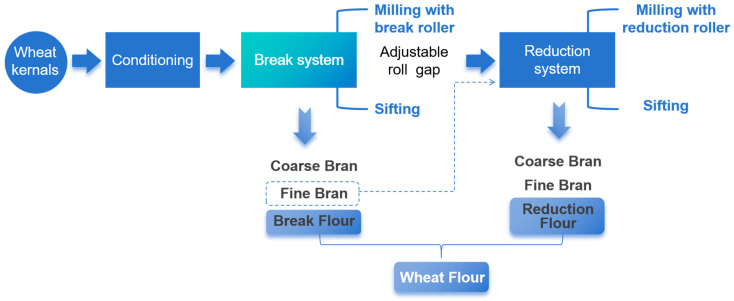
Process flow chart of the laboratory-scale wheat milling system.

This study focused on the regulation of the break system to produce wheat flours with different processing degrees. As illustrated in Figure 1, the control group (CL) underwent standard milling with the following roll gap settings: 0.5 mm coarse crushing and 0.05 mm fine stripping in the break system, as well as 0.05 mm and 0.03 mm in the reduction system. To investigate processing effects, two modified approaches were implemented, as follows: (1) the double break milling (2BM) method maintained identical parameters to the CL group, but incorporated an additional grinding pass (total passes = 2); (2) the double sifting (2S) method introduced an extra sifting step (total sifting passes = 2) in the break system to prolong material–sieve contact time. Furthermore, to examine roll gap influences, three comparative treatments were established by increasing the second-stage roll gap (IRG) from 0.05 mm to 0.08 mm in the CL, 2BM, and 2S processes, designated as CL-IRG, 2BM-IRG, and 2S-IRG, respectively. All processing procedures were performed in triplicate to obtain experimental wheat flour samples.

### 2.3. Analysis of Wheat Flour Properties

The flour extraction rate was calculated as the ratio of the flour weight obtained after milling to the initial wheat weight. Protein and ash contents were determined following the standard AACC Methods 46-30.01 [17] and 08-01.01 [18], respectively. All measurements were performed in triplicate.

The particle size distribution of wheat flour was measured using a laser diffraction analyzer (Nano ZS90, Malvern Instruments Ltd., Worcestershire, UK). The particle sizes corresponding to 10%, 50%, and 90% cumulative distribution in the system were recorded as D10, D50, and D90, respectively, with the volume mean diameter represented as D(4,3). D10, D50, and D90 represent the fine, median, and coarse fractions of the flour’s particle size distribution, respectively. The test was repeated three times.

### 2.4. Preparation of Microwave Freeze-Dried NFINs

The preparation of fresh noodles began with mixing water and wheat flour of different processing degrees (34:100, *w*/*w*) to form dough crumbs, followed by conditioning at 25 °C and 50% relative humidity for 30 min. The conditioned dough was then processed through a noodle machine using the following stepwise reduction protocol: initial sheeting at 3.0 mm thickness, followed by three successive folding and sheeting cycles at reduced thicknesses of 2.0 mm, 1.5 mm, and 1.0 mm. Finally, the dough sheet was cut into noodles measuring 2.5 mm in width and 1.0 mm in thickness. This standardized procedure was applied consistently for both refined and moderately processed noodles.

NFINs refer to instant noodle products that are dehydrated through microwave freeze-drying. Exactly 100 g of noodles was cooked in boiling water until complete gelatinization (no white core visible), the cooking process took approximately 4 ± 0.5 min. The noodles were immediately cooled in room-temperature water for 10 s and were then portioned (300 g per sample) into dishes for freezing at −25 °C for at least 24 h. The frozen noodles were dried using a custom-designed microwave freeze-drying apparatus developed by our research team; detailed descriptions of the equipment configuration are available in our previously published paper [19]. The drying parameters were as follows: a condenser temperature of −40 °C, a vacuum pressure of 200 Pa, and a microwave power density of 1.5 W/g. The drying process was terminated when a constant weight was achieved.

For clarity in description, the NFINs produced from wheat flour obtained through different milling processes (CL, 2S, 2BM, CL-IRG, 2S-IRG, and 2BM-IRG) will be referred to as CL noodles, 2S noodles, 2BM noodles, CL-IRG noodles, 2S-IRG noodles, and 2BM-IRG noodles. This standardized nomenclature will be consistently used throughout subsequent discussions to facilitate the comparison of noodle characteristics across different milling processes.

### 2.5. Determination of the Quality Characteristics of NFINs

#### 2.5.1. Color

The color of dried NFINs was measured using a colorimeter (Color i5, X-Rite Incorporated, Grand Rapids, MI, USA). The whiteness index (WI) was calculated according to the following formula:

(1) $$\mathrm{WI}=100\;-\sqrt{\left[{\left(100-L^{*}\right)}^{2}+{a^{*}}^{2}+{b^{*}}^{2}\right]}$$

where L^*^ represents the lightness or brightness of the color, a^*^ indicates the red–green chromaticity (positive values denote a redder hue, while negative values indicate a greener hue), and b^*^ represents the yellow–blue chromaticity (positive values indicate a yellower tone, whereas negative values suggest a bluer tone). Measurements were repeated five times.

#### 2.5.2. Rehydration Ratio

The dried noodles were immersed in a water bath at 100 °C until the white center inside the noodles disappeared. The rehydration ratio was expressed as the mass ratio before and after cooking. Three independent replicates were conducted.

#### 2.5.3. Cooking Loss

The cooking loss of the dried noodles was measured following the method of Liu et al. [20] and was calculated as the ratio of the residue mass in the cooking water to the initial noodle mass. The test was performed in triplicate.

#### 2.5.4. Texture Properties

The texture properties of the cooked NFINs were determined according to the method described by Wei et al. [21] with slight modifications. Five noodle strands were placed parallel on the testing platform and a compression test was performed using a P36/R probe (Stable Micro Systems Ltd., Godalming, UK). The pre-speed, post-speed, and test speed were all set at 1 mm/s. Each sample was compressed twice with a compression ratio of 70%, an interval of 5 s between compressions, and a trigger force of 5 g. The results were calculated as the average of six measurements.

#### 2.5.5. Microstructure Observation

The microstructure of dried NFIN cross-sections was observed using a scanning electron microscope (SEM, TM3030, Hitachi High-Technologies Corporation, Tokyo, Japan). Prior to observation, the samples were sputter-coated with gold to enhance conductivity. SEM images were then acquired at an acceleration voltage of 15 kV with a magnification of 300×.

#### 2.5.6. Water State Distribution

The NFINs were soaked in 100 °C water until the white center inside the noodles disappeared. The moisture state and distribution of the cooked NFINs were analyzed using a low-field nuclear magnetic resonance (LF-NMR) analyzer (NMI20-015V-I, Niumag Analytical Instruments Co., Suzhou, China) with the Carr–Purcell–Meiboom–Gill (CPMG) pulse sequence. The measurement parameters were set as follows: sampling points = 100,028, 90° pulse time = 6.5 μs, 180° pulse time = 12 μs, number of echoes = 1000, echo time = 0.4 ms, spectral width = 100 kHz, and waiting time = 4000 ms. The transverse relaxation time (*T*_2_) spectra were obtained by processing the CPMG decay curves using the Simultaneous Iterative Reconstruction Technique (SIRT) algorithm in the NMR analysis software (v1.0, Niumag Electric Co., Shanghai, China). Three independent replicates were conducted.

### 2.6. In Vitro Starch Digestibility

The in vitro starch digestion of cooked NFINs was performed according to the method described by Englyst et al. [22]. The total starch content and glucose content were determined using a starch content assay kit (BC0700, 50T/48S, Solarbio Science & Technology Co., Ltd., Beijing, China) and a glucose oxidase–peroxidase (GOPOD) assay kit (G0504F, Suzhou Grace Biotechnology Co., Ltd., Suzhou, China), respectively. The hydrolysis ratio of starch (HRS) was calculated using the following equation [23]:

(2) $$\mathrm{HRS}\;\left(\%\right)\;=\;\frac{{(m}_{1}\times \;0.9)}{m}\;\times \;100\%$$

where *m* is the total starch content (mg) and *m*_1_ represents the glucose equivalents at sampling points (mg).

The hydrolysis index (HI) and expected glycemic index (eGI) were calculated as follows [24]:

(3) $$\mathrm{HI}\;=\;{\mathrm{AUC}}_{1}/{\mathrm{AUC}}_{0}$$

(4) eGI=0.549HI+39.71

where AUC_1_ denotes the area under the hydrolysis curve of the test sample; AUC_0_ indicates the area under the hydrolysis curve of white bread—0.54939.71 (reference control). Three independent replicates were conducted.

### 2.7. Organoleptic Assessment

Healthy and taste-sensitive panelists were selected, excluding individuals with color blindness, rhinitis, stomatitis, or gastroenteritis. Sensory training was conducted, covering basic sensory knowledge, common evaluation methods, and analysis, as well as scoring criteria. Twenty randomly chosen evaluators (with a 1:1 male-to-female ratio) participated in the sensory assessment of the noodles. NFINs from different groups were cooked in boiling water, drained, and placed in separate containers labeled with random codes. To minimize errors, all evaluators rinsed their mouths with water between tasting different noodle formulations. A 100-point hedonic scale was used to evaluate the overall acceptability, including hardness (20 points), chewiness (20 points), smoothness (20 points), elasticity (20 points), and flavor (20 points).

### 2.8. Statistical Analysis

All experiments were performed, at least, in triplicate. The results are expressed as mean ± standard deviation (SD). Statistical analyses were conducted using Origin software (v2022, OriginLab Corporation, Northampton, MA, USA) and SPSS software (version 19.0, SPSS Inc., Chicago, IL, USA). One-way analysis of variance (ANOVA) followed by Duncan’s multiple range test was employed for statistical evaluation, with a statistical significance level of *p* < 0.05. A two-tailed Pearson correlation analysis was conducted to examine the correlation among the variables.

## 3. Results and Discussion

### 3.1. Flour Extraction Rate and Proximate Composition

The flour extraction rates of different milling processes are shown in Table 1. The 2BM-IRG process achieved the highest extraction rate, significantly surpassing other groups (*p* < 0.05). This indicated that the synergistic effect of increasing break grinding passages (2BM) and increasing the roll gap (IRG) can effectively improve the extraction rate. Compared to the CL group, the 2BM group showed a 1.22% increase in extraction rate, which was attributed to the retention of finer bran particles through multiple break grinding steps. The 2S group exhibited no significant difference in extraction rate compared to the control (*p* > 0.05), suggesting that additional sifting steps did not affect the flour extraction rate. Notably, merely increasing the roll gap (CL-IRG) led to a 2.44% reduction in extraction rate. This result was associated with incomplete endosperm separation, where residual endosperm was expelled along with the bran. However, the highest extraction rate in the 2BM-IRG group demonstrated that additional break milling steps can compensate for the negative impact of a wider roll gap. The reason for this was the secondary grinding opportunity that was provided to under-processed bran–endosperm composites in subsequent break milling stages after initial incomplete separation due to the expanded roll gap.

The protein content of wheat flour exhibited significant variations among different milling processes (Table 1). Both the 2BM and 2BM-IRG groups showed a significantly higher protein content compared to the CL group (*p* < 0.05), which can be explained through two mechanisms. First, according to wheat kernel structural distribution theory, proteins and minerals are predominantly concentrated in the bran and aleurone layers [25]. The 2BM process, through multiple break grinding steps, more effectively liberated these protein-rich components (as evidenced by its higher flour extraction rate), aligning with findings from Pojić et al. [26] suggesting that the protein content in break system flour increased with additional grinding passages. Second, during later milling stages, mechanical action promoted a more complete dissociation of protein complexes in the germ, further elevating protein levels [27]. In contrast, neither the 2S process (2S and 2S-IRG groups) nor the CL-IRG process demonstrated significant effects on protein content relative to the CL group (*p* > 0.05).

The impact of different flour milling processes on ash content is shown in Table 1. The 2BM-IRG group exhibited the highest ash content, significantly surpassing other treatment groups (*p* < 0.05). This result aligned with the trend observed in flour extraction rate. According to the structural distribution characteristics of wheat kernels, mineral elements are primarily concentrated in the aleurone layer and bran tissues [28]. The 2BM process enhanced ash content by retaining more fine bran particles and aleurone layers through repeated break milling. Notably, the 2S-IRG group showed the lowest ash content, which was attributed to the loss of fine mineral particles during the sieving process.

### 3.2. Particle Size of Wheat Flour

The particle size distribution analysis (D10, D50, D90) and volume mean diameter (D(4,3)) of wheat flours obtained through different milling processes showed significant variations (Table 2). Compared to the CL process, the 2S process resulted in significant reductions in D10, D50, D90, and D(4,3) values (*p* < 0.05), which is primarily attributed to its increased sifting times. The enhanced shearing action from double sifting promoted the breakdown of larger particles, leading to increased proportions of fine flour. In contrast, the 2BM process caused significant decreases in D10 and D50 values (*p* < 0.05), while showing no significant change in D90 (*p* > 0.05). This pattern reflected the progressive grinding effect of double break milling, which improved the yield of fine particles, while having limited secondary grinding effects on already-liberated starch granules. Consequently, the D(4,3) values showed no significant difference to those of the CL group (*p* > 0.05).

When the roll gap was increased, significant increases were observed in D10, D50, D90, and D(4,3) values (*p* < 0.05). This effect of roll gap enlargement was consistent with findings reported by Bojanić et al. [29]; this was related to the reduced unit area pressure in increased roll gaps, which weakened endosperm fragmentation. However, under specific conditions (e.g., D50 and D90 in the 2S-IRG group, as well as D50 in the 2BM-IRG group), no significant differences were observed (*p* > 0.05). The D(4,3) values of flours produced by different processes ranged from 65.27 to 95.04 μm, aligning with reported values (61–95 μm) for typical wheat flour particle sizes [30].

### 3.3. Color, Rehydration Ratio, and Cooking Loss of NFINs

**Figure 2:**
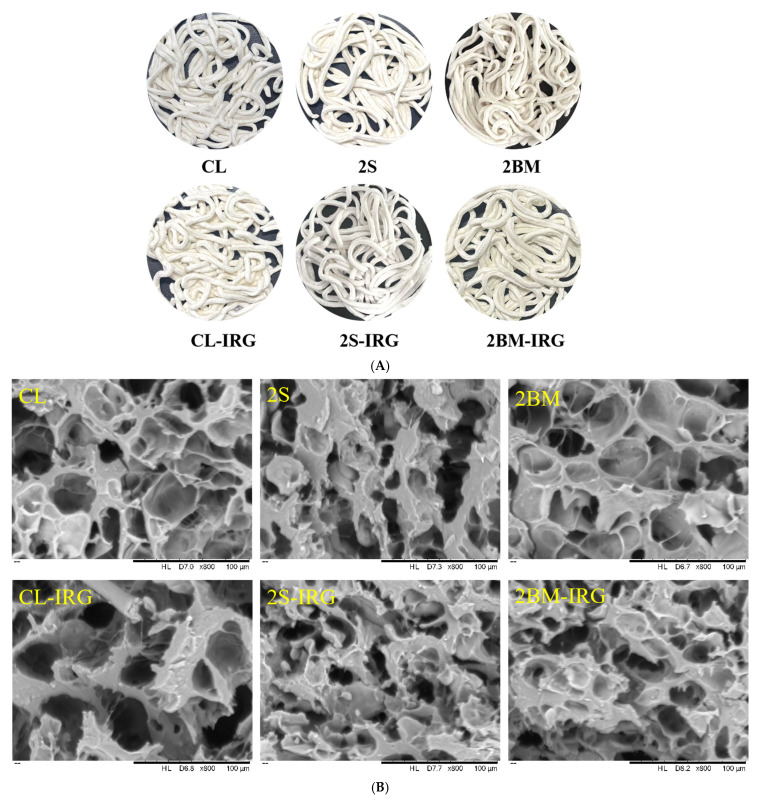
The appearance (**A**) and microstructure (**B**) of NFINs. CL (control): standard milling process; 2S (double sifting): CL process with an additional sifting pass (total passes = 2); 2BM (double break milling): CL process with an extra break milling pass (total passes = 2); CL-IRG/2BM-IRG/2S-IRG: corresponding processes (CL/2BM/2S) with an increased second-stage roll gap.

Color is a key indicator for evaluating noodle quality and serves as the most intuitive criterion for consumers to assess food quality. The appearance and color of NFINs produced from wheat flour obtained through different milling processes are shown in Figure 2A and Table 3, respectively. The whiteness index (WI) represents the overall color of noodles [31]. The color parameters (L^*^, a^*^, b^*^, and WI values) of NFINs prepared from wheat flour processed by different milling methods showed no significant differences (*p* > 0.05), indicating that the milling process does not affect the overall color of NFINs. Even though the 2BM process incorporated more wheat bran into the flour, it did not result in poorer color. This phenomenon can be explained by two key factors. First, the increased bran content was insufficient to alter the overall color. Second, the repeated break milling process effectively reduced bran particle size, with these finer particles exhibiting reduced light scattering properties, which consequently minimized color variations.

The rehydration capacity of NFINs showed significant variations (*p* < 0.05) among different milling processes (Table 3). The 2BM-IRG noodles exhibited the highest rehydration ratio. This phenomenon could be attributed to an increased pentosan content. As the number of break passages increased (2BM and 2BM-IRG processes), the intensified grinding action more effectively disrupted the cell wall structure of wheat kernels, facilitating the release of soluble pentosans from the bran and aleurone layer [32]. Research has demonstrated that water-soluble pentosans possess an exceptional water-holding capacity, as their hydroxyl groups can form hydrogen bond networks with water molecules. This mechanism was further supported by our experimental data—the 2BM-IRG process not only achieved the highest rehydration ratio in NFINs but also showed a significantly greater flour extraction rate (69.80%) and ash content (0.54%) compared to other groups, confirming the more efficient extraction of pentosan-rich aleurone and bran components. Additionally, existing study indicated that increased break passages (2BM and 2BM-IRG processes) resulted in a higher damaged starch content [27]. The exposed internal structure of damaged starch enhances its ability to bind with water molecules [29]. Notably, although the CL-IRG group also adjusted roll gap settings, it demonstrated the lowest rehydration ratio due to the absence of synergistic effects from multi-stage break milling.

The cooking loss is a critical parameter for assessing the cooking quality of instant noodles. It varied significantly (*p* < 0.05) depending on milling processes (Table 3), with 2BM-IRG noodles exhibiting the highest cooking loss, while 2S-IRG noodles exhibited the lowest cooking loss. This variation stemmed from the following two key mechanisms: (1) increased break passages in the 2BM-IRG process elevated damaged starch content, whose water-induced disintegration during cooking exacerbated loss, and (2) finer bran fragments from repeated break milling released more water-soluble pentosans and phenolics. In contrast, the additional sieving in the 2S-IRG process selectively removed soluble fines while preserving endosperm integrity, thereby showing optimal cooking stability.

### 3.4. Water State and Distribution in NFINs

**Figure 3:**
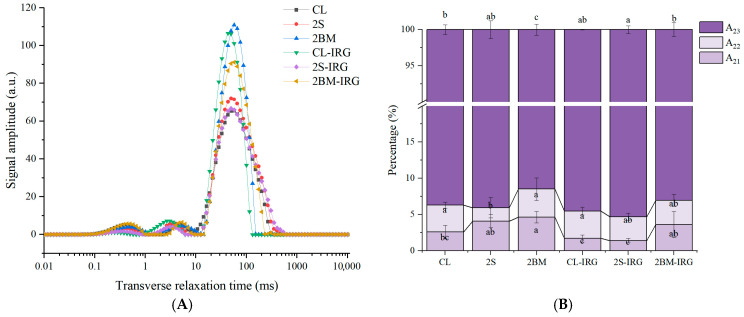
*T*_2_ profiles (**A**) and water state distribution (**B**) in cooked NFINs. CL (control): standard milling process; 2S (double sifting): CL process with an additional sifting pass (total passes = 2); 2BM (double break milling): CL process with an extra break milling pass (total passes = 2); CL-IRG/2BM-IRG/2S-IRG: corresponding processes (CL/2BM/2S) with an increased second-stage roll gap. Different lowercase letters indicate significant differences at *p* < 0.05.

The water state and distribution of cooked NFINs serves as a critical indicator reflecting product quality and processing characteristics. Variations in the flour composition induced by different milling processes may lead to changes in the water state and distribution of the prepared NFINs, consequently affecting noodle quality. As shown in Figure 3A, three distinct water states exist in cooked NFINs—strongly bound water (*T*_21_, 0.1–1 ms), weakly bound water (*T*_22_, 1–10 ms), and free water (*T*_23_, >10 ms) [33]. Their corresponding peak area ratios to the total area are denoted as *A*_21_, *A*_22_, and *A*_23_, respectively. The strongly bound water is tightly associated with gluten proteins, starch, and other macromolecules; the weakly bound water resides in the protein network and starch molecular pores with limited mobility; the free water exists outside the flour macromolecules with the highest degree of freedom [34,35].

NFINs prepared from 2BM and 2BM-IRG flours exhibited a higher strongly bound water content (Figure 3B) (*A*_21_) due to two synergistic mechanisms. Firstly, the double break milling in these processes more thoroughly disrupted bran cell walls, significantly increasing the release of soluble arabinoxylans from the aleurone layer—these polysaccharides contain abundant hydroxyl groups (-OH) that form extensive hydrogen bonding networks with water molecules. Secondly, the elevated protein content enhanced the water-binding capacity through both hydrophilic amino acid residues and the formation of a more continuous gluten network.

NFINs prepared from CL-IRG and 2S-IRG flours showed a lower strongly bound water content (*A*_21_), primarily due to two key factors related to milling modifications. First, the increased roll gap led to the insufficient disruption of bran cell walls, limiting the release of water-binding arabinoxylans from the aleurone layer. Second, the coarser milling resulted in larger starch granules with a reduced surface area, providing fewer active sites for water binding. These combined effects—incomplete cell wall breakdown and diminished starch granule accessibility—significantly impaired the flour’s water retention capacity. The 2S-IRG process further exacerbated this issue through excessive sieving, which removed finer particles that typically contribute to water absorption.

### 3.5. Textural Properties of NFINs

**Figure 4:**
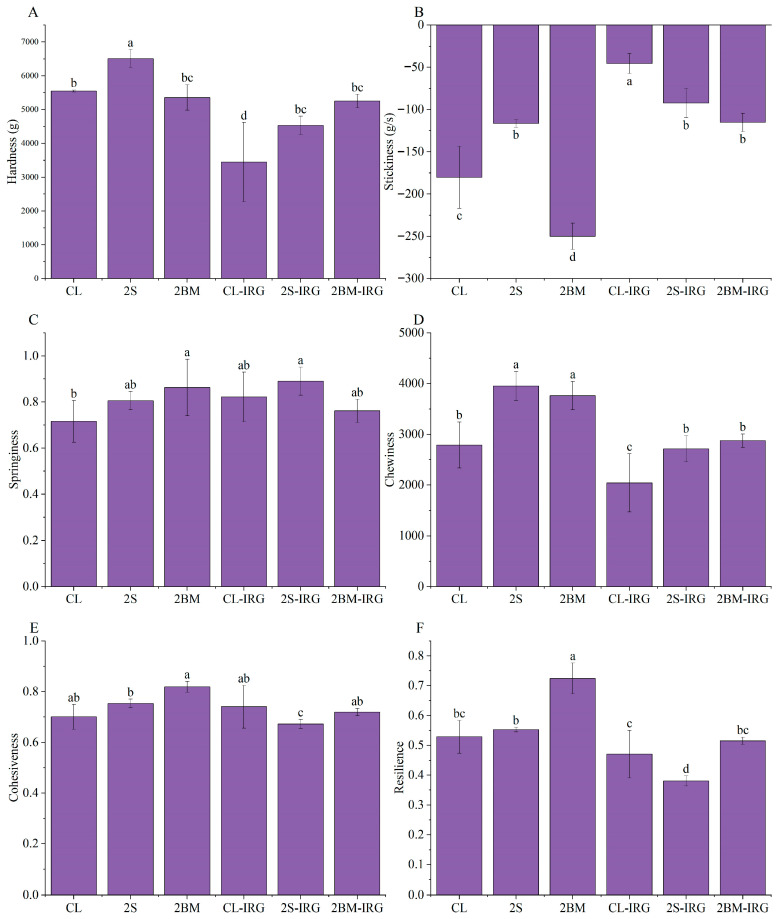
Textural properties of NFINs made from wheat flour obtained by different milling processes: (**A**) Hardness; (**B**) Stickiness; (**C**) Springiness; (**D**) Chewiness; (**E**) Cohesiveness; (**F**) Resilience. CL (control): standard milling process; 2S (double sifting): CL process with an additional sifting pass (total passes = 2); 2BM (double break milling): CL process with an extra break milling pass (total passes = 2); CL-IRG/2BM-IRG/2S-IRG: corresponding processes (CL/2BM/2S) with an increased second-stage roll gap. Different lowercase letters indicate significant differences at *p* < 0.05.

The quantitative evaluation of textural properties in cooked noodles serves as a reliable indicator of sensory quality [36]. The results demonstrated significant variations (*p* < 0.05) in the textural properties of NFINs produced through different milling processes (Figure 4). Specifically, 2S noodles exhibited the highest hardness and chewiness, while 2BM noodles displayed the lowest adhesiveness and highest resilience. These differences primarily stemmed from how milling processes modulate flour’s physicochemical properties. The 2S process, through additional sieving, generated flour and incorporated bran with smaller particle sizes. These finer particles not only strengthened the binding force with the gluten matrix but also maintained the integrity of the gel network by reducing cooking loss. This was aligned with findings from Patra et al. [37] regarding bran particle size effects. Notably, the 2BM flour’s higher protein content increased the gluten network crosslinking density while reducing starch leaching, ultimately showing its distinctive texture properties of reduced adhesiveness and enhanced resilience. The 2BM-IRG noodles presented balanced textural parameters; while its elevated protein content promoted gluten network formation, the high bran retention (evidenced by increased ash content) simultaneously disrupted the gluten continuum. This opposite effect resulted in the final formed gluten network having a moderate strength compared to other treatments. From a quality perspective, ideal noodles should possess a moderate hardness (providing sufficient chewing resistance without excessive rigidity) and an appropriate chewiness (offering pleasant mastication without being laborious), along with excellent elasticity and a suitable cohesiveness. These emphasize that optimal noodle quality depends not on the extreme values of individual parameters but rather on their balanced integration, which should be evaluated in conjunction with sensory analysis.

### 3.6. Sensory Evaluation

**Figure 5:**
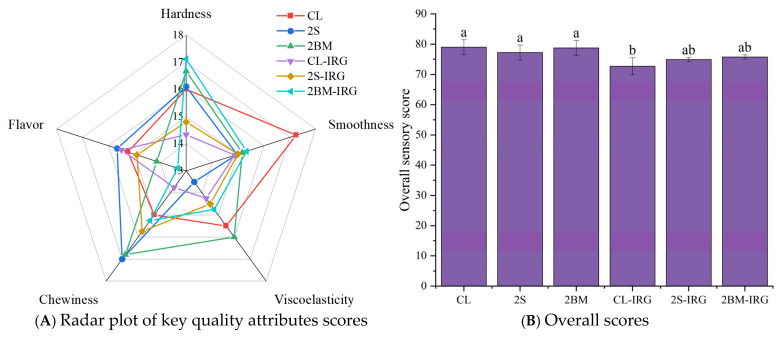
The sensory evaluation of NFINs made from wheat flour obtained by different milling processes. (**A**) Scores of key quality attributes. (**B**) Overall scores. CL (control): standard milling process; 2S (double sifting): CL process with an additional sifting pass (total passes = 2); 2BM (double break milling): CL process with an extra break milling pass (total passes = 2); CL-IRG/2BM-IRG/2S-IRG: corresponding processes (CL/2BM/2S) with an increased second-stage roll gap. Different lowercase letters indicate significant differences at *p* < 0.05.

The consumer acceptance of NFINs was evaluated based on key sensory attributes including hardness, elasticity, smoothness, chewiness, and flavor; the results are presented in Figure 5. Significant differences (*p* < 0.05) were observed in the overall sensory scores of NFINs produced from wheat flour processed through different milling processes. The CL noodles achieved the highest overall score (79.0 ± 2.4), though not significantly different from 2S (77.2 ± 2.8) and 2BM (78.8 ± 1.9) noodles, while the CL-IRG group showed markedly reduced scores (*p* < 0.05). The 2BM-IRG noodles achieved an intermediate overall sensory score.

Scores from the individual sensory indicators revealed distinct process-dependent characteristics. The 2S noodles demonstrated significantly improved smoothness and chewiness scores, attributable to the refined bran particle size achieved through additional sieving stages. This particle size reduction minimized surface roughness while enhancing structural homogeneity. In contrast, the 2BM noodles exhibited a superior elasticity, which can be attributed to their enhanced protein content, facilitating the formation of an optimized gluten network structure (as verified via SEM images). The 2BM-IRG noodles achieved the highest hardness score in sensory evaluation, which was related to their moderate hardness level. However, the flavor score of 2BM-IRG noodles was compromised, which is likely due to the cumulative loss of key volatile compounds during intensive milling operations.

### 3.7. Microstructure

Figure 2B presents the SEM images of NFINs prepared from wheat flours of different sources. The interior of the noodles exhibited a typical honeycomb-like structure, which was caused by the sublimation of frozen water inside the noodles during the microwave freeze-drying process. This was aligned with Yin’s [13] findings on freeze-dried starch systems. The CL noodles had dense internal pores and thin pore walls, with a high degree of gelatinization, making it difficult to distinguish starch granules from the protein matrix. This was consistent with the findings reported by Gallo et al. [38], where pasta showed almost indistinguishable starch granules and protein matrix due to highly coagulated protein and gelatinized starch. The 2S noodles had thicker pore walls and a more stable structure, resulting in a greater hardness in texture (as shown in Section 3.4). The 2BM noodles displayed a uniform and continuous network structure with a smooth surface and no accumulation of fine particles. This structural feature corresponded to their lower viscosity and higher resilience.

The microstructure continuity of CL-IRG noodles reduced, which was due to the insufficient release of aleurone layer-binding proteins and the decreased adsorption of starch granule interface proteins, which collectively weaken the network structure [39]. The larger pore size in the microstructure of CL-IRG noodles contributed to their low hardness, as the porous structure can buffer mechanical stress. Compared to CL-IRG noodles, 2S-IRG and 2BM-IRG noodles exhibited a more compact microstructure. This structural difference could be attributed to their distinct compositional characteristics. In the case of 2BM-IRG flour, its higher protein content enhanced gluten network formation, resulting in a denser structure [40]. Meanwhile, 2S-IRG flour contains less bran residue, which was beneficial since the insoluble fiber present in bran could physically hinder gluten cross-linking. Sun et al. [41] demonstrated that dough without wheat bran exhibits a more compact gluten network structure. Consequently, the reduced bran content effectively minimized its interference with the development of a continuous gluten network.

### 3.8. In Vitro Starch Digestibility of NFINs

**Figure 6:**
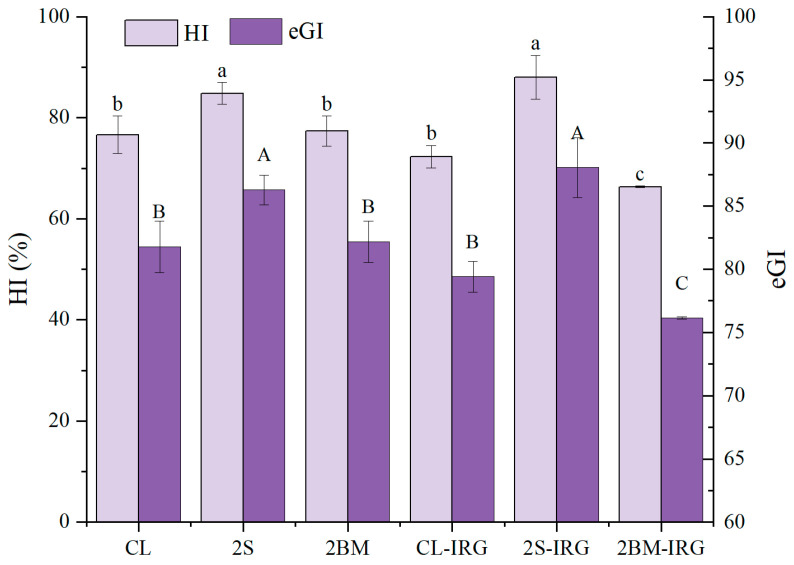
The HI and eGI values of NFINs made from wheat flour obtained by different milling processes. CL (control): standard milling process; 2S (double sifting): CL process with an additional sifting pass (total passes = 2); 2BM (double break milling): CL process with an extra break milling pass (total passes = 2); CL-IRG/2BM-IRG/2S-IRG: corresponding processes (CL/2BM/2S) with an increased second-stage roll gap. Different capital letters indicate significant differences (*p* < 0.05) in HI. Different lowercase letters indicate significant differences (*p* < 0.05) in eGI.

The estimated glycemic index (eGI) and hydrolysis index (HI) are indicators of a food’s digestibility, with higher values indicating an easier digestibility and vice versa. Figure 6 presents the eGI and HI values of NFINs prepared from wheat flour obtained through different milling processes. The 2BM-IRG group showed the lowest eGI and HI values (*p* < 0.05), which may be attributed to its unique compositional and structural characteristics. Although starch is the primary component of noodles, other factors such as interactions between starch and non-starch components in the noodle matrix can also influence digestibility [42]. The double break milling process in the 2BM-IRG group increased the content of non-starch components like glucans and polyphenols in the wheat flour. These components delay digestion by forming complexes with starch or by competitively inhibiting α-amylase activity [43,44]. In contrast, the 2S and 2S-IRG groups exhibited significantly higher eGI and HI values than the CL group (*p* < 0.05). This could be explained by (1) the additional sifting removing bran particles and consequently reducing digestion-resistant components, and (2) the increased friction between starch and sieves during additional sifting, which increased the damaged starch content and facilitated α-amylase adsorption and the hydrolysis of α-1,4 glycosidic bonds, thereby enhancing digestibility [45]. Interestingly, despite employing the same 2S process, the 2S-IRG group showed significantly lower eGI and HI values than the 2S group (*p* < 0.05). This difference was due to the increased roll gap enlarging the starch granule size and reducing digestibility, which is consistent with previous studies on the relationship between starch granule size and digestibility characteristics [46]. Notably, the 2BM and CL-IRG groups showed no significant differences in eGI and HI values compared to the CL group (*p* > 0.05), indicating that neither increasing the number of break milling process nor enlarging the roll gap alone significantly affects starch digestibility. These findings demonstrate that different milling processes can modulate starch digestibility through distinct mechanisms, with the 2BM-IRG process being particularly effective in producing noodles with a lower glycemic response.

### 3.9. Correlation Analysis

**Figure 7:**
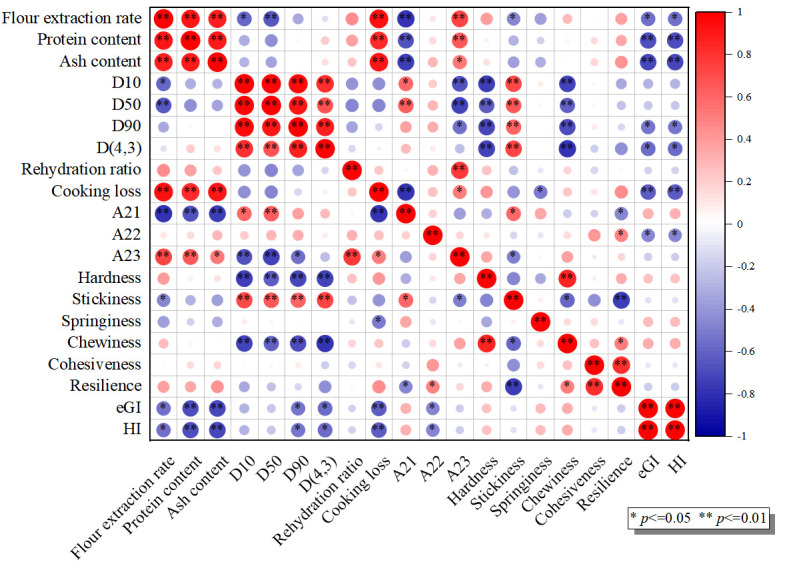
Pearson’s correlation of wheat flour properties and the quality of NFINs.

A correlation analysis between wheat flour properties and the quality of NFINs was performed; the results are shown in Figure 7. The eGI and HI values exhibited highly significant negative correlations (*p* < 0.01) with the protein content and ash content of wheat flour, while showing a significant negative correlation (*p* < 0.05) with the D(4,3) volume mean diameter. These findings indicated that adjusting the milling process—such as reducing processing precision to increase protein and ash content, while enlarging particle size—can significantly decrease the starch digestibility of NFINs. Furthermore, eGI and HI values were also highly negatively correlated with cooking loss, which is consistent with the findings reported by Zou et al. [47]. The loss of soluble components (e.g., partial starch and proteins) during cooking may reduce starch accessibility to digestive enzymes, thereby lowering its hydrolysis rate. However, in contrast to Zou et al.’s [42] study, no significant correlation was observed between starch digestibility and textural properties (e.g., hardness and elasticity). This suggested that under the experimental conditions of this study, the chemical composition (e.g., protein and ash content) of wheat flour played a more critical role in starch digestibility than its physical structure (e.g., gluten network).

The hardness and chewiness of noodles showed a highly significant negative correlation with flour particle size distribution parameters (*p* < 0.01). As the particle size of flour decreases, its specific surface area increases significantly. This change accelerates the water absorption rate of starch granules, enhances gelatinization, and consequently promotes the formation of a more compact gel network structure [37], ultimately endowing the noodles with a firmer texture and an improved chew resistance. The observed strong positive correlations between cooking loss, flour extraction rate, and protein/ash content (*p* < 0.01) primarily result from three mechanistic pathways: First, flours with higher extraction rates inherently contain more bran fragments and aleurone layer materials. These components physically interfere with continuous gluten network development during dough formation [41]. Second, while total protein content increased, the proportion of functional gluten proteins may decrease relative to non-structural proteins like albumins and globulins, leading to a reduced water-binding capacity (demonstrated by NMR studies showing higher free water populations). Third, an elevated ash content serves as a marker of bran contamination, with the introduced dietary fibers and minerals creating hydrophilic sites that compete with starch for water molecules while simultaneously introducing structural defects that facilitate component leaching during cooking [48].

## 4. Conclusions

This study systematically elucidates the regulatory mechanisms of different milling processes on the physicochemical properties of wheat flour, as well as on the quality of microwave freeze-dried instant noodles. Through modifications in flour composition (protein and ash content) and physical characteristics (particle size distribution), various milling techniques significantly influenced the quality attributes of microwave freeze-dried noodles. The 2BM-IRG combined process not only enhanced flour protein and ash content but also endowed the noodles with optimal rehydration properties and the lowest starch digestibility, albeit with the highest cooking loss. The 2S process improved noodle texture by refining particle size, while the 2BM process enhanced elasticity through protein network formation. Notably, reducing processing precision (increasing ash and protein content) effectively inhibited starch digestion (significantly lower eGI values in the 2BM-IRG group). By precisely controlling milling parameters under laboratory conditions, this study reveals the intrinsic regulatory mechanisms through which milling processes affect the physicochemical properties of wheat flour, as well as the quality and nutritional characteristics of microwave freeze-dried instant noodles. This provides a theoretical foundation and milling process optimization strategies for developing specialized noodle flours tailored to specific market demands. Based on the established relationships between milling processes and end-product qualities, we recommend the following targeted applications: for premium instant noodles requiring high elasticity, e.g., hot pot noodles and ramen, the 2BM process is recommended; for health-conscious products demanding low starch digestibility, e.g., meal replacement noodles, the 2BM-IRG combined process is advised. However, it is important to recognize that this study was conducted using a laboratory-scale milling system; thus, its conclusions may have limitations when translated to large-scale industrial production. Based on these findings, future research should focus on pilot-scale validation and industrial production line adaptation.

## Figures and Tables

**Table 1:** The flour extraction rate, protein content, and ash content of wheat flour obtained by different milling processes.

Sample	Flour Extraction Rate (%)	Protein Content (%)	Ash Content (%)
CL	66.46 ± 0.68 ^c^	10.33 ± 0.06 ^c^	0.49 ± 0.02 ^bc^
2S	65.63 ± 0.23 ^cd^	10.26 ± 0.04 ^c^	0.46 ± 0.03 ^bc^
2BM	67.68 ± 0.29 ^b^	10.66 ± 0.05 ^b^	0.51 ± 0.01 ^ab^
CL-IRG	64.02 ± 0.89 ^d^	10.30 ± 0.02 ^c^	0.47 ± 0.03 ^bc^
2S-IRG	64.89 ± 0.12 ^d^	10.25 ± 0.02 ^c^	0.45 ± 0.01 ^c^
2BM-IRG	69.80 ± 0.03 ^a^	10.98 ± 0.01 ^a^	0.54 ± 0.02 ^a^

Different superscript letters in the same column indicate significant differences at *p* < 0.05. CL (control): standard milling process; 2S (double sifting): CL process with an additional sifting pass (total passes = 2); 2BM (double break milling): CL process with an extra break milling pass (total passes = 2); CL-IRG/2BM-IRG/2S-IRG: corresponding processes (CL/2BM/2S) with an increased second-stage roll gap.

**Table 2:** The particle size distribution of wheat flour obtained by different milling processes.

Samples	D10 (μm)	D50 (μm)	D90 (μm)	D(4,3) (μm)
CL	5.32 ± 0.03 ^c^	38.12 ± 0.39 ^b^	178.62 ± 2.21 ^c^	69.18 ± 0.38 ^d^
2S	5.12 ± 0.08 ^d^	36.51 ± 0.91 ^c^	171.23 ± 0.42 ^d^	65.27 ± 0.82 ^e^
2BM	5.06 ± 0.05 ^d^	34.85 ± 0.11 ^d^	177.36 ± 1.75 ^c^	67.33 ± 0.77 ^de^
CL-IRG	6.83 ± 0.06 ^a^	49.43 ± 1.29 ^a^	234.43 ± 0.76 ^a^	95.04 ± 0.33 ^a^
2S-IRG	5.42 ± 0.06 ^b^	36.59 ± 0.45 ^c^	173.49 ± 3.92 ^d^	76.42 ± 1.39 ^c^
2BM-IRG	5.26 ± 0.03 ^c^	34.71 ± 0.13 ^d^	190.03 ± 0.92 ^b^	86.38 ± 2.20 ^b^

Different superscript letters in the same column indicate significant differences at *p* < 0.05. CL (control): standard milling process; 2S (double sifting): CL process with an additional sifting pass (total passes = 2); 2BM (double break milling): CL process with an extra break milling pass (total passes = 2); CL-IRG/2BM-IRG/2S-IRG: corresponding processes (CL/2BM/2S) with an increased second-stage roll gap.

**Table 3:** Physical properties of NFINs made from wheat flour obtained by different milling processes.

Sample	L^*^	a^*^	b^*^	*WI*	Rehydration Ratio	Cooking Loss (%)
CL	88.32 ± 1.70 ^a^	0.07 ± 0.06 ^a^	8.87 ± 0.27 ^a^	85.14 ± 1.38 ^a^	2.62 ± 0.5 ^ab^	6.18 ± 0.68 ^bc^
2S	87.40 ± 4.00 ^a^	0.16 ± 0.17 ^a^	8.69 ± 0.68 ^a^	84.62 ± 3.71 ^a^	2.32 ± 0.42 ^ab^	5.74 ± 0.59 ^bcd^
2BM	87.88 ± 1.80 ^a^	0.15 ± 0.07 ^a^	9.32 ± 0.66 ^a^	83.65 ± 1.11 ^a^	2.45 ± 0.58 ^ab^	6.54 ± 0.11 ^ab^
CL-IRG	85.15 ± 1.93 ^a^	0.19 ± 0.09 ^a^	8.48 ± 0.59 ^a^	84.05 ± 0.22 ^a^	1.85 ± 0.16 ^b^	5.17 ± 0.63 ^cd^
2S-IRG	86.37 ± 0.07 ^a^	0.06 ± 0.03 ^a^	8.29 ± 0.31 ^a^	84.45 ± 1.83 ^a^	2.53 ± 0.63 ^ab^	4.71 ± 0.13 ^d^
2BM-IRG	87.75 ± 2.74 ^a^	0.18 ± 0.16 ^a^	9.41 ± 0.57 ^a^	82.89 ± 1.93 ^a^	2.82 ± 0.62 ^a^	7.60 ± 0.57 ^a^

Different superscript letters in the same column indicate significant differences at *p* < 0.05. CL (control): standard milling process; 2S (double sifting): CL process with an additional sifting pass (total passes = 2); 2BM (double break milling): CL process with an extra break milling pass (total passes = 2); CL-IRG/2BM-IRG/2S-IRG: corresponding processes (CL/2BM/2S) with an increased second-stage roll gap.

## Data Availability

The original contributions presented in the study are included in the article, further inquiries can be directed to the corresponding authors.
